# High Glucose Increases Lactate and Induces the Transforming Growth Factor Beta-Smad 1/5 Atherogenic Pathway in Primary Human Macrophages

**DOI:** 10.3390/biomedicines12071575

**Published:** 2024-07-16

**Authors:** Kareem Awad, Laura Kakkola, Ilkka Julkunen

**Affiliations:** 1 Institute of Biomedicine, Faculty of Medicine, University of Turku, 20520 Turku, Finland; laura.kakkola@utu.fi (L.K.); ilkka.julkunen@utu.fi (I.J.); 2 Medical Faculty, Ruprecht-Karls-University of Heidelberg, 69117 Heidelberg, Germany; 3 Academy of Scientific Research & Technology (ASRT-STARS), Cairo 11516, Egypt; 4 Institute of Pharmaceutical and Drug Industries Research, National Research Centre, Giza 12622, Egypt; 5 Clinical Microbiology, Turku University Hospital, 20521 Turku, Finland; 6 InFLAMES Research Flagship, University of Turku, 20014 Turku, Finland

**Keywords:** high glucose, macrophage, TGFβ1, Smad, diabetes, atherosclerosis

## Abstract

Hundreds of millions of people worldwide are expected to suffer from diabetes mellitus. Diabetes is characterized as a dynamic and heterogeneous disease that requires deeper understanding of the pathophysiology, genetics, and metabolic shaping of this disease and its macro/microvascular complications. Macrophages play an essential role in regulating local immune responses, tissue homeostasis, and disease pathogenesis. Here, we have analyzed transforming growth factor beta 1 (TGFβ1)/Smad signaling in primary human macrophages grown in normal (NG) and high-glucose (HG; +25 mM glucose) conditions. Cell culture lactate concentration and cellular phosphofructokinase (PFK) activity were increased in HG concentrations. High glucose levels in the growth media led to increased macrophage mRNA expression of TGFβ1, and TGFβ-regulated HAMP and PLAUR mRNA levels, while the expression of TGFβ receptor II remained unchanged. Stimulation of cells with TGFβ1 protein lead to Smad2 phosphorylation in both NG and HG conditions, while the phosphorylation of Smad1/5 was detected only in response to TGFβ1 stimulation in HG conditions. The use of the specific Alk1/2 inhibitor dorsomorphin and the Alk5 inhibitor SB431542, respectively, revealed that HG conditions led TGFβ1 to activation of Smad1/5 signaling and its downstream target genes. Thus, high-glucose activates TGFβ1 signaling to the Smad1/5 pathway in primary human macrophages, which may contribute to cellular homeostasis in a harmful manner, priming the tissues for diabetic complications.

## 1. Introduction

To date, diabetes mellitus accounts more than 500 million cases worldwide and is considered a chronic and heterogeneous group of diseases. Although certain disease mechanisms of diabetes are known, deeper understanding of the pathophysiology, genetics and metabolic disorders contributing to the disease and especially influencing diabetes-related macro/microvascular complications is urgently needed [1].

Macrophages are innate immune cells that reside in many organs and orchestrate various physiological and pathological processes in humans [2,3,4,5,6]. Macrophages are classified into M1 or M2 types based on physiological conditions: infection-induced M1 macrophages protect against pathogens, while cytokine-differentiated M2 macrophages are involved in tissue repair [3,4,5]. Abnormal function of these immune cells can contribute to chronic inflammation and subsequent pathological consequences including cardiometabolic disorders [3,4,5,6]. The function of macrophages is regulated through signaling pathways induced by various extracellular stimuli.

Transforming growth factor-β1 (TGFβ1) is a multifunctional cytokine that, based on environmental or cellular context, leads to a diversity of cellular responses, including cell proliferation, differentiation, migration, apoptosis, and cancer progression [7,8]. TGFβ1 binds to the cytoplasmic serine/threonine kinase domain of type II receptor which assembles a receptor I complex. TGFβ1 binding to its receptor complex leads to phosphorylation of Smad family proteins, which translocate into the nucleus and control gene expression [9,10]. Smad proteins are classified into three groups: receptor-regulated (R-Smad; Smad1, Smad2, Smad3, Smad5, and Smad8/9), inhibitory (I-Smad; Smad6 and Smad7), and common-mediator (Co-Smad; Smad4) [10] Smad proteins. I-Smads can inhibit the activation of R-Smads by competing with R-Smad to bind to its receptor and/or by recruiting specific ubiquitin ligases to the activated receptor complex [9,10].

TGFβ1 has been shown to initiate differential gene expression in immune cells, based on the type of environmental stimuli and expressed Smads, via TGFβ1/Smad2/3 or TGFβ1/Smad1/5 signaling [3]. The balance between Smad1/5- and Smad2/3-dependent signaling, and the expression of inhibitory Smad7, could define the outcome of the effect of TGFβ1 on macrophages, and differential gene expression could lead to either protective or harmful health consequences [3]. The TGFβ1-induced genes that are associated with atherogenesis include HAMP and PLAUR [3]. 

HAMP is a gene coding for hepcidin that regulates iron hemostasis in macrophages [11]. High hepcidin levels result in iron trapping in macrophages, and retention of iron in macrophages leads to the development of foam cells and plaque formation that enhance atherosclerosis [12,13,14]. PLAUR is a gene coding for urokinase plasminogen activator surface receptor (uPAR) that is elevated in atherosclerosis and in many cancer types [15,16,17,18,19]. Particularly, increased uPAR modulates monocyte functions and enhances atherogenesis [15,20]. 

Metabolic events such as high glucose or increased tissue lactate levels have been shown to affect TGFβ1/Smad signaling (reviewed in ref. [21]). Our aim here was to investigate the effect of high glucose on the expression of atherogenic genes via TGFβ1/Smad signaling to provide further knowledge on the possible role of high glucose in the development of atherosclerosis.

## 2. Materials and Methods

### 2.1. Primary Human Macrophage Culture

Monocytes were isolated from buffy coat blood products from healthy blood donors, and macrophages were differentiated from monocytes according to a standard procedure [3,22]. Buffy coat preparations were purchased from either the German or Finnish Red Cross Blood Service, Mannheim, Germany or Helsinki, Finland. In brief, peripheral blood mononuclear cells were purified from 13 buffy coat preparations using Ficoll density gradient followed by either CD14^+^ magnetic cell sorting (Miltenyi Biotech, Bergisch Gladbach, Germany) or by allowing mononuclear cells to adhere to plates (1 × 10^6^ cells per mL; Falcon; Becton Dickinson, Franklin Lakes, NJ, USA) for 1 h in RPMI 1640 medium (Sigma-Aldrich, St. Louis, MO, USA) at +37 °C. Macrophage serum free culture medium (Marcophage-SFM, Thermo Fisher Scientific, Waltham, MA, USA) was supplemented with penicillin/streptomycin, 20 mM HEPES, and 2 mM l-glutamine. Non-adherent cells were removed by washing with cold phosphate-buffered saline (PBS). The adherent monocytes were differentiated into macrophages by growing them in Macrophage-SFM adjusted to levels of normal (~17.5 mM) and high (+25 mM resulting in 42.5 mM) D-glucose (Sigma-Aldrich, USA) before being supplemented with penicillin/streptomycin, 20 mM HEPES, 2 mM l-glutamine, and human recombinant granulocyte macrophage colony-stimulating factor (GM-CSF; 10 ng/mL; Nordic Biosite, Täby, Sweden). The cells were differentiated into macrophages for 7 days with a change of fresh culture medium every 2 days and monitoring of differentiated macrophages via light microscopy. Alternatively, monocytes were differentiated to M2 type macrophages by stimulating with IL-4 (10 ng/mL; Peprotech, London, UK) in X-vivo 10 serum-free medium (Cambrex, Verviers, Belgium) for 6 days. TGF-β1 (TEBU Peprotech, Frankfurt am Main, Germany) was used at a final concentration of 10 ng/mL. Dexamethasone (Sigma-Aldrich, Munich, Germany) was used at a concentration of 100 nM. Inhibitors of signaling pathways (dorsomorphin or SB431542) and inhibitors of ALK1/2 or ALK5 were used in 1 µM or 5 µM concentrations, respectively. Supernatants of culture media and cell lysates (cells scraped off carefully with rubber cell scratchers into appropriate reagent, see below) were collected and stored at −80 °C until further analysis.

### 2.2. Lactate Assay 

Lactate was assayed colorimetrically in the supernatants of normal and +25 mM glucose culture of 2 × 10^6^ cells using the Abcam lactate assay kit (ab65331, Cambridge, UK) according to the manufacturer’s instructions. After 30 min of incubation at room temperature, the lactate concentration was measured using a multi-well plate (VICTOR^®^ Nivo, PerkinElmer, Waltham, MA, USA) reader at 450 nm and calculated using a lactate standard curve.

### 2.3. Phosphofructokinase (PFK) Assay

PFK1 activity was assayed colorimetrically from cell lysates of macrophages cultured in a normal and +25 mM glucose concentration using the 6-phosphofructokinase activity assay kit from Abcam (ab155898, UK). Cell lysates were prepared by lysing 10^6^ cells with the lysis buffer provided in the kit, according to the protocol provided by the manufacturer. PFK activity was measured in the cell lysates using a multi-well plate reader (VICTOR^®^ Nivo, PerkinElmer) at 450 nm at two time points, kinetic T1 (20 min) and T2 (40 min). An NADH standard curve was used to calculate PFK1 activity.

### 2.4. Quantitative Real-Time Reverse Transcriptase PCR (qRT-PCR) 

Total cellular RNA from the macrophages (2 × 10^6^ cells) cultured in normal and +25 mM glucose concentrations was extracted using a PureLink^®^ RNA Mini Kit (Invitrogen, Waltham, MA, USA) according to the protocol of the manufacturer. RNA was quantified with a nano-spectrophotometer (DeNovix, Wilmington, DE, USA). Equal concentrations (100 ng) of RNA were reverse-transcribed into cDNA using the High-Capacity cDNA Reverse Transcription Kit (Thermo Fisher Scientific, USA) according to the manufacturer’s protocol. To amplify individual genes, specific primers for TGFβ1, Smad7, HAMP and PLAUR were used (Table 1). RT-qPCR was performed using 100 ng cDNA and PowerTrack SYBR Green Kit (Thermo Fisher Scientific, USA) according to the manufacturer’s protocol with Rotor Gene Q PCR machine (Qiagen; Hilden, Germany). The RT-qPCR program was as follows: initial heat activation at 95 °C for 15 min, followed by 40 cycles of denaturation at 94 °C for 15 s, annealing at 52 °C for 30 s, and extension at 72 °C for 30 s. Levels of specific mRNA expression were normalized to mRNA expression levels of GAPDH, and the relative change in gene expression was calculated according to formula 2^−∆∆Ct^.

### 2.5. Analysis of the Phosphorylation of Smads Proteins

For the analysis of the phosphorylation of Smads proteins, isolated monocytes were cultured under normal and high glucose concentrations (+25 mM) and differentiated into M2 type macrophages with IL-4 for 6 days. In addition, TGFβ1 receptor expression was induced in the cells with simultaneous incubation with dexamethasone for 6 days [23]. To induce the signaling pathway, TGFβ1 (10 ng/mL) was added for 3 h. Subsequently, 1–3 × 10^6^ cells were lysed in 50 μL lysis buffer (50 mM Tris HCl, pH 7.4, 150 mM NaCl, 1 mM EDTA, 5 mM NaF, 1% NP-40, 2 mM NaVO_3_, 0.25% Na deoxycholate, and 1× Complete protein inhibitors; Roche, Mannheim, Germany). Western blotting (WB) was performed as previously described [12]. Briefly, the samples were separated on a 12% PAGE and transferred to a nitrocellulose membrane. For the detection of proteins, rabbit anti-human Smad2/3 mAb, anti-human Smad1 mAb or anti-human phospho-Smad2 mAb or anti-human phospho-Smad1/5 mAb (Cell Signaling Technology, Danvers, MA, USA) was used in a 1:1000 dilution. As a secondary antibody, HRP-linked anti-rabbit IgG whole A (GE Healthcare, Chicago, IL, USA) was used in a dilution of 1:5000. Chemiluminescence detection was performed using SuperSignal Pico peroxidase substrate (Pierce, Waltham, MA, USA), and exposed to Kodak BioMax light films for 1 to 30 min. Films were developed in an X-ray film processor.

### 2.6. Flow Cytometry

Cells were scraped carefully from a 6-well plate into 15 mL tubes. The suspension was centrifuged at 300× *g* for 8 min in a swing-out rotor. The supernatant was aspirated, and cells were washed once in 5 mL of 2% BSA in PBS and centrifuged. For staining, the cell pellet was re-suspended in 2% BSA in PBS (approximately 10^6^ cells in 100 µL). Then, 5 µL of anti-human TGFβRII phycoerythrin conjugated mouse IgG antibody (R&D systems) or PE conjugated mouse IgG1 K isotype control (BD Bioscience, Franklin Lakes, NJ, USA) was added to the cell suspension (1:60) and incubated on ice for 1 h in the dark. The suspension was centrifuged for 10 min at 200× *g* at +4 °C. Supernatants were discarded, and cells were washed two times with 2% BSA in PBS. After the last centrifugation, the supernatant was aspirated and cell pellets were suspended in 350 µL of 2% BSA in PBS. Staining was analysed using BD FACS Canto II (BD Bioscience, USA).

### 2.7. Statistical Analysis

Data were analyzed using the Graph Pad Prism software version 8 (La Jolla, San Diego, CA, USA) and Microsoft Excel 2013. Normality was determined by the Shapiro–Wilk test. Data are presented as mean ± SD. Statistical significance was assessed by either a paired Student’s *t*-test to compare normal to high-glucose groups from the same donors or by an ordinary one-way ANOVA followed by Tukey’s multiple comparisons test to assess significant difference between the donors. A *p*-value of less than 0.05 was considered statistically significant.

## 3. Results

### 3.1. Lactate Concentration and Phosphofructokinase Enzyme Activity in Macrophages Cultured under Normal or High Glucose Levels

**Figure 1:**
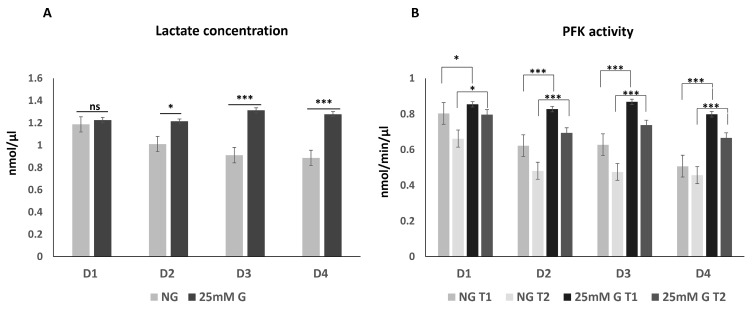
(**A**) Lactate concentration (nmol/µL) and (**B**) phosphofructokinase (PFK) enzyme activity (nmol/min/µL) in culture medium of macrophages from four donors (D1–D4) grown for 7 days in normal (NG) and in high (25 mM) glucose concentrations. Error bars represent the measurement results of duplicates or triplicates, * *p* < 0.05, *** *p* < 0.001).

Macrophages from four donors were grown in normal and in high (+25 mM) glucose, and lactate concentration was measured on day 7 (Figure 1A). When comparing macrophages of the same donor grown in normal glucose to macrophages grown in high glucose, the lactate production in macrophages grown in high glucose was significantly increased (in 3 out of 4 donors). Phosphofructokinase (PFK) activity was measured on day 7 (Figure 1B). According to the PFK activity assay kit, activity was measured at two time points (20 min (T1) and 40 min (T2)). Similarly, the PFK activity was significantly increased after culturing the macrophages in high glucose compared to the normal glucose. The results indicate that the high-glucose environment causes metabolic changes in the macrophages, with increased PFK activity and higher lactate concentrations.

### 3.2. The Expression of TGFβ1, Smad7, HAMP, and PLAUR mRNA in Macrophges Cultured under Normal or High-Glucose Conditions

**Figure 2:**
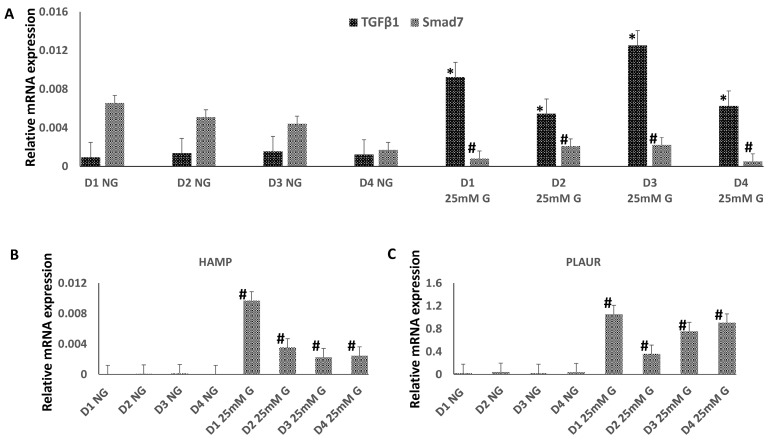
Relative expression levels of (**A**) TGFβ1 and Smad7, (**B**) HAMP, and (**C**) PLAUR mRNAs in the macrophages from four donors (D1–D4) grown in normal (NG) and in high (+25 mM) glucose concentrations. Error bars represent the measurements in duplicates or triplicates, * or # refer to statistical difference *p* < 0.05 between the samples of the same donor in normal glucose (NG) and high glucose (+25 mM) concentrations.

The relative expression of four mRNAs, TGFβ1, Smad7, HAMP and PLAUR, was quantified on day 7 in the macrophages grown in normal and in high (+25 mM) glucose (Figure 2). GAPDH mRNA was used as a reference. The expression of TGFβ1 was significantly increased in high glucose, compared to normal glucose, in macrophages obtained from all four donors (Figure 2A). However, the expression of SMAD7 was significantly decreased in high glucose concentrations. The expression levels of both HAMP (Figure 2B) and PLAUR mRNA (Figure 2C) were significantly increased in high glucose compared to normal glucose in macrophages obtained from all four donors. The results were similar within the donors, although variation in expression levels between the donors was evident.

### 3.3. TGFβ1/Smad Signaling in Macrophages Cultured under Normal or High Glucose

**Figure 3:**
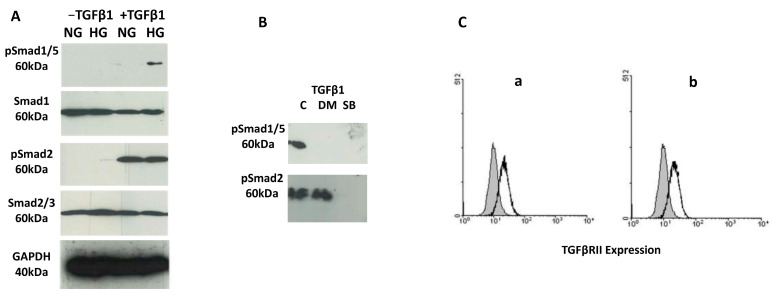
TGFβ1/Smad signaling in macrophages (**A**) cultured under a normal (NG) or high glucose (HG) concentration, and induced with TGFβ1. Phosphorylation of Smad proteins was analyzed with immunoblotting. Phosphorylated Smad1/5 and Smad2 and total Smad1/5 and Smad2/3 proteins were detected. GAPDH was used as a loading control. (**B**) Dorsomorphin (DM), a specific inhibitor of ALK1/2, or SB431542 (SB), a specific inhibitor of ALK5, was added 1 h before TGFβ1 stimulation, and the phosphorylation of Smad proteins in high-glucose conditions was analyzed with immunoblotting. (**C**) FACS analysis of the expression of TGFβRII on the surface of macrophages cultured under normal (a) and high (b)-glucose conditions. (*n* = 5; tests were performed in duplicate).

To analyze the phosphorylation events specific to the TGFβ1 signaling pathway, isolated monocytes were cultured in normal or high-glucose conditions and differentiated into TGFβ1-receptor-expressing macrophages with IL-4 and dexamethasone. The TGFβ1 signaling pathway was activated with TGFβ1, and the cells were analyzed for the phosphorylation of Smad proteins (Figure 3). Smad2 was phosphorylated upon TGFβ1 stimulation in both normal and high-glucose conditions (Figure 3A), while phosphorylation of Smad1/5 was observed only in high-glucose conditions. Of note, variation between the donors was observed with differential intensities of phosphorylated Smads (Table 2, Supplementary Figures S1–S4).

To further analyze the TGFβ1 signaling pathway, specific inhibitors for receptor serine/threonine kinases ALK1/2 and ALK5 (dorsomorphin and SB431542, respectively) were used in high-glucose conditions to inhibit the activation of these receptors in the presence of TGFβ1 (Figure 3B). A specific ALK5 inhibitor, SB431542, (SB, 5 µM) inhibits phosphorylation of both Smad2 and Smad1/5, while the specific ALK1/2 inhibitor, dorsomorphin, inhibits the phosphorylation of Smad1/5 but not that of Smad2. This indicates that TGFβ1-induced phosphorylation of Smad2 requires only the activation of ALK5 receptor, while the phosphorylation of Smad1/5 requires the recruitment of the ALK2 receptor in addition to ALK5.

To confirm the expression of the TGFβ1 receptor, TGFβRII, on the surface of macrophages, TGFβRII expression was analyzed by FACS on macrophages cultured in normal and high-glucose conditions (Figure 3C). The expression level of TGFβRII was not affected by the increasing glucose concentration. These results indicate that macrophages cultured in normal or high glucose concentrations express equal amounts of TGFβRII, and thus the differential effect of TGFβ1 induction on the phosphorylation of Smad proteins is not dependent on the expression of the receptor but rather on downstream signaling molecules regulated by different glucose concentrations.

## 4. Discussion

Diabetes mellitus is a disease which may appear with characteristic clinical symptoms like polydipsia, polyuria, weight loss, or ketoacidosis-associated symptoms. The disease may also appear as a non-symptomatic form without its typical clinical symptoms (which are defined as a constant increase in blood glucose levels [24]), meaning that recently, high blood glucose levels have come into question as the only important parameter for defining diabetes. Diabetes mellitus, including type 1 and type 2 diabetes, probably comprises several distinct disorders of the metabolism or post-translational mechanisms, which evidences the need to create novel approaches for understanding the disease and its complications [25]. Late diabetic complications including retinopathy, nephropathy, neuropathy, and other macro/micro vascular complication raises the need to develop in vitro cell culture models to better understand the disease’s pathogenesis in different tissues.

In the present work, we cultured human primary macrophages in normal and high-glucose conditions and analyzed cellular responses to high glucose levels. We adjusted normal and high glucose for the cultured macrophage population based on the viability of macrophages to glucose concentrations found in diabetes. There is now evidence that different cell types tolerate specific glucose concentrations in vitro before they show significant changes due to hyperglycemia or abnormal glucose concentration in their growth culture [26,27]. In our cell system, we clearly observed increased gene expression of TGFβ1 and HAMP and PLAUR genes (higher mRNA levels). It is thus likely that constitutively high glucose levels may also enhance the production of TGFβ1 and its downstream target genes HAMP and PLAUR in human primary macrophages. Interestingly, high glucose levels did not regulate TGFβRII expression, suggesting that the effect of high glucose in our experimental system is mediated at the TGFβ signaling level. TGFβ1 is an important regulator of immune tolerance and homeostasis whose inhibition may contribute to the eradication of cancer and cardiovascular deteriorations [28,29]. TGFβ1 levels have been demonstrated to be increased in experimental atherosclerosis [30], aortic aneurysmal disease [31], atherosclerotic plaques in patients with coronary hearth disease [32], and in monocytes circulating in patients with atrial fibrillation and fibrosis [33].

**Figure 4:**
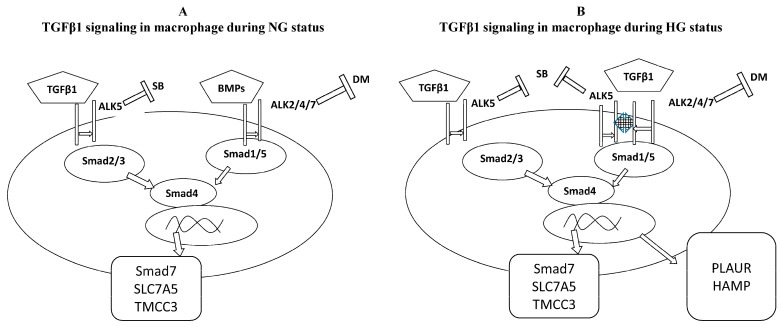
Schematic model of the action of TGFβ1/Smad signaling in human macrophages cultured in normal (NG) vs. high (HG)-glucose conditions. (**A**) Under normal glucose conditions, TGFβ1 binds to a type II receptor which assembles to receptor ALK5, leading to phosphorylation of the Smad2 and 3 proteins. Phosphorylation of Smad1 and 5 proteins occurs through BMPs binding to their specific receptor and recruitment of ALK2. Subsequently, these events lead to TGFβ1- or BMP-induced gene expression, respectively. (**B**) Under high-glucose conditions, TGFβ1 expression increases, and binding of TGFβ1 to a type II receptor leads to activation of both ALK5 and ALK2 receptors, leading to phosphorylation of both Smad2 and 3 as well as Smad1 and 5. Smad1 and 5 activation subsequently leads to differential gene expression, including atherosclerotic genes HAMP and PLAUR. Abbreviations: TGFβ1, transforming growth beta 1; BMPs, bone morphogenic proteins; SB, SB431542; DM, dorsomorphin.

As TGFβ-activated signaling molecules, Smad proteins consist of two domains, mad homology 1 (MH1) and MH2, which are responsible for DNA binding and protein interactions with other regulatory proteins, respectively [10]. This suggests that various functional domains are responsible for the flexible and differential functions of Smad family proteins. The present work shows that hyperglycemia in human macrophages changes the balance between Smad1/5 and Smad2/3-dependent signaling. This may contribute to the outcome of the effect of TGFβ1 on cardiovascular risks, where Smad1/5 is responsible for the expression of pro-atherogenic genes and Smad2/3 downregulates TGFβ1 functions by upregulating Smad7 expression. Smad7 is known to downregulate signaling by other Smad proteins (10). Interestingly, we saw a great variation in TGFβ1-induced Smad activation among individual blood donors. Even though there was some variation between the donors, the general trend in differentiated macrophages obtained from the monocytes of a total of seven blood donors was very similar. In high-glucose conditions, TGFβ1, HAMP, and PLAUR mRNA expression was increased, and TGFβ1 activated Smad1/5 phosphorylation (in addition to Smad2) suggesting a constant biological phenomenon among all analyzed donors. Thus, this work provides evidence on the cell receptor regulation of this possible Smad1/5–TGFβ1 atherogenic pathway, where inhibition of Alk1/2 but not Alk5 by pharmacological inhibitors may block the activation of the Smad1/5 signaling pathway (Figure 4).

The potentially harmful effects of high glucose levels on the Smad1/5 pathway have been associated with the expression of HAMP and PLAUR genes. Accumulation of iron has been detected in atherosclerotic lesions, particularly in macrophages/foam cells. However, the exact mechanisms explaining the role of hepcidin in atherogenesis and how it induces iron retention in plaque macrophages have never been clearly elucidated [12,13,14]. In the present study, we provide a possible mechanism for enhanced HAMP expression, i.e., high glucose levels led to enhanced Smad1/5 signaling triggered by increased lactate levels, or a “Warburg”-like status enhanced the expression of HAMP in hyperglycemic macrophages.

Here, we demonstrated increased PLAUR expression in hyperglycemic conditions. PLAUR has been associated with the regulation of uPAR in a mechanism including vascular endothelial growth factor, interfering with the blood–retinal barrier that characterizes the early stages of vascular dysfunction in diabetes [34]. Higher serum uPAR levels were associated with higher mortality beyond traditional risk factors among African Americans with type 2 diabetes mellitus [35]. Higher serum levels of uPAR are also an independent and significant indicator of cardiovascular events in type 1 diabetes [36,37]. Our data and previously published studies suggest that the Smad1/5 PLAUR pathway is a mechanism underlying the association of high glucose levels with risk of cardiovascular events, which may yield new molecular targets for the development new diagnostic/therapeutic strategies.

Our data agree with previous observations that the role of increased lactate levels and PFK activity, which are seen in hyperglycemia, is associated with immunometabolic regulation of TGFβ1 expression and perhaps that of other immunoregulatory molecules [38,39,40]. A high-glucose environment will activate a genetic cascade that can polarize macrophages toward a specific M1-like phenotype, and this could lead to further tissue damage in diabetic conditions, a phenomenon which is, however, a matter for further investigation [41,42,43].

In summary, our study provides evidence that high glucose in cultured primary human macrophages increases the lactate concentration and upregulates the expression of TGFβ1 and its downstream HAMP and PLAUR genes. TGFβ1 in high-glucose conditions can also activate Smad 1/5 phosphorylation, likely contributing to enhanced HAMP and PLAUR mRNA expression. Our study may provide a mechanistic explanation for increased glucose levels in primary human cells activating TGFβ1 signaling pathways and thus contributing to pathogenic events in hyperglycemic diseases. This information may be helpful and important in the further development of drugs that can reduce the appearance of severe diabetes-associated disease complications.

## 5. Conclusions

The present work provides new knowledge on the role of high glucose in activating TGFβ1 signaling to the Smad1/5 pathway in primary human macrophages and presents one possible explanation of the role of HAMP and PLAUR in immune and vascular deteriorations, which are the main complications of diabetes. Moreover, it suggests a possible method of terminating this pathway by designing pharmacological interventions specific to the TGFβ family receptor type.

## Figures and Tables

**Table 1:** List of primers.

Gene	Primers Forward/Reverse	Accession Number
TGFβ1	TTATTGAGCACCTTGGGCAC	NM_000660.7
	TCTCTGGGCTTGTTTCCTCAC	
Smad7	TTCCTCGGAAGTCAAGAGGCT	NM_005904.4
	CCATCGGGTATCTGGAGTAA	
HAMP	ACAACTTGCAGAGCTGCAAC	NM_021175.4
	GCAGCAGAAAATGCAGATGG	
PLAUR	TGAAGATCACCAGCCTTACC	NM_002659.4
	TGATGAGCCACAGGAAATGC	
GAPDH	GGTGGTCTCCTCTGACTTCAAC	NM_002046.7
	GTTGCTGTAGCCAAATTCGTTG	

**Table 2:** The arbitrary intensity of phosphorylation of Smad2 and Smad1/5 upon stimulation with TGFβ1 in macrophages from seven individual donors. The differences in intensity (i.e., protein expression in immunoblotting) show the differential responses of donors under the same conditions.

Donor	pSmad2	pSmad1/5
1	LG: ++ HG: +++	LG: −− HG: ++
2	LG: ++ HG: ++	LG: −− HG: ++
3	LG: +++ HG: +++	LG: −− HG: +++
4	LG: ++ HG: ++	LG: −− HG: +
5	LG: +++ HG: +++	LG: −− HG: ++
6	LG: +++ HG: +++	LG: −− HG: +
7	LG: +++ HG: −	LG: −− HG: +++

(+++; highest expression, ++; higher expression, +; expressed, −−; not expressed).

## Data Availability

All data are available from the corresponding author upon reasonable request. The original contributions presented in this study (blotting/protein analysis) are included in the Supplementary Materials as a minimal dataset necessary for evaluation. Further inquiries can be directed to the corresponding author/s. The numeric data set is presented as figures and included within the manuscript and can be made available by the authors in other forms upon request for presentation protection rights.

## Supplementary Materials

The following supporting information can be downloaded at: https://www.mdpi.com/article/10.3390/biomedicines12071575/s1, Figures S1–S6: TGFβ1 signaling through Smads in macrophages cultured under NG or HG conditions in analyzed donors under the same experimental conditions.

## Abbreviations

TGFβ1	transforming growth factor beta 1
NG	normal glucose
HG	high glucose
PFK	phosphofructokinase
uPAR	urokinase plasminogen activator surface receptor
GM-CSF	granulocyte-MQ colony-stimulating factor
PBS	phosphate-buffered saline
