# Supplementary material for: High Glucose Increases Lactate and Induces the Transforming Growth Factor Beta-Smad 1/5 Atherogenic Pathway in Primary Human Macrophages
Source: Biomedicines. 2024 Jul 16;12(7):1575. doi: 10.3390/biomedicines12071575 (PMC11275184; doi:10.3390/biomedicines12071575)
Supplement: Supplementary file 1 [file biomedicines-12-01575-s001.zip › biomedicines-3111257-supplementary.pdf]

Figure S1

MS Figure 3A (pSmad1,5)

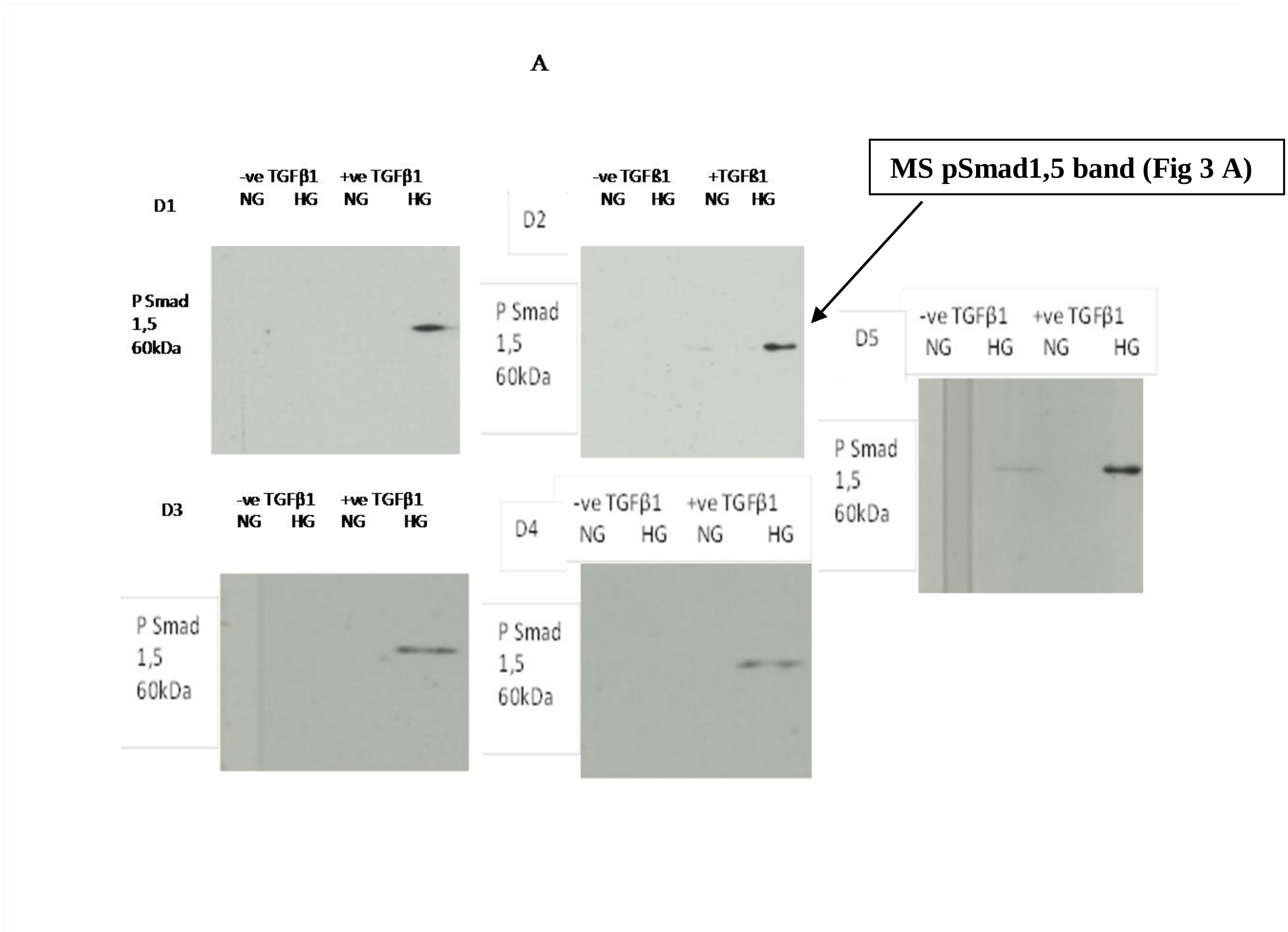

Figure S1. TGFβ1 signaling through Smad 1, 5 in Macrophages cultured under NG or HG in 5 Donors (D1-D5) under the same experimental conditions:

Monocytes were cultured in both NG or HG conditions and stimulated 6 days with IL4/dex.TGFβ1 (10ng/ml) was added. After incubation, the cells were harvested and lysed in a lysis buffer supplemented with phosphatase and protease inhibitors. WB was performed using specific first antibody for the phospho smad 1, 5. WB shows the phosphorylation of smad1, 5 observed only with Macrophages under our defined *in vitro* HG system. The difference in band intensities (protein expressions) shows the different donors responses within the same experimental environment.

Figure S2

MS Figure 3A (pSmad2)

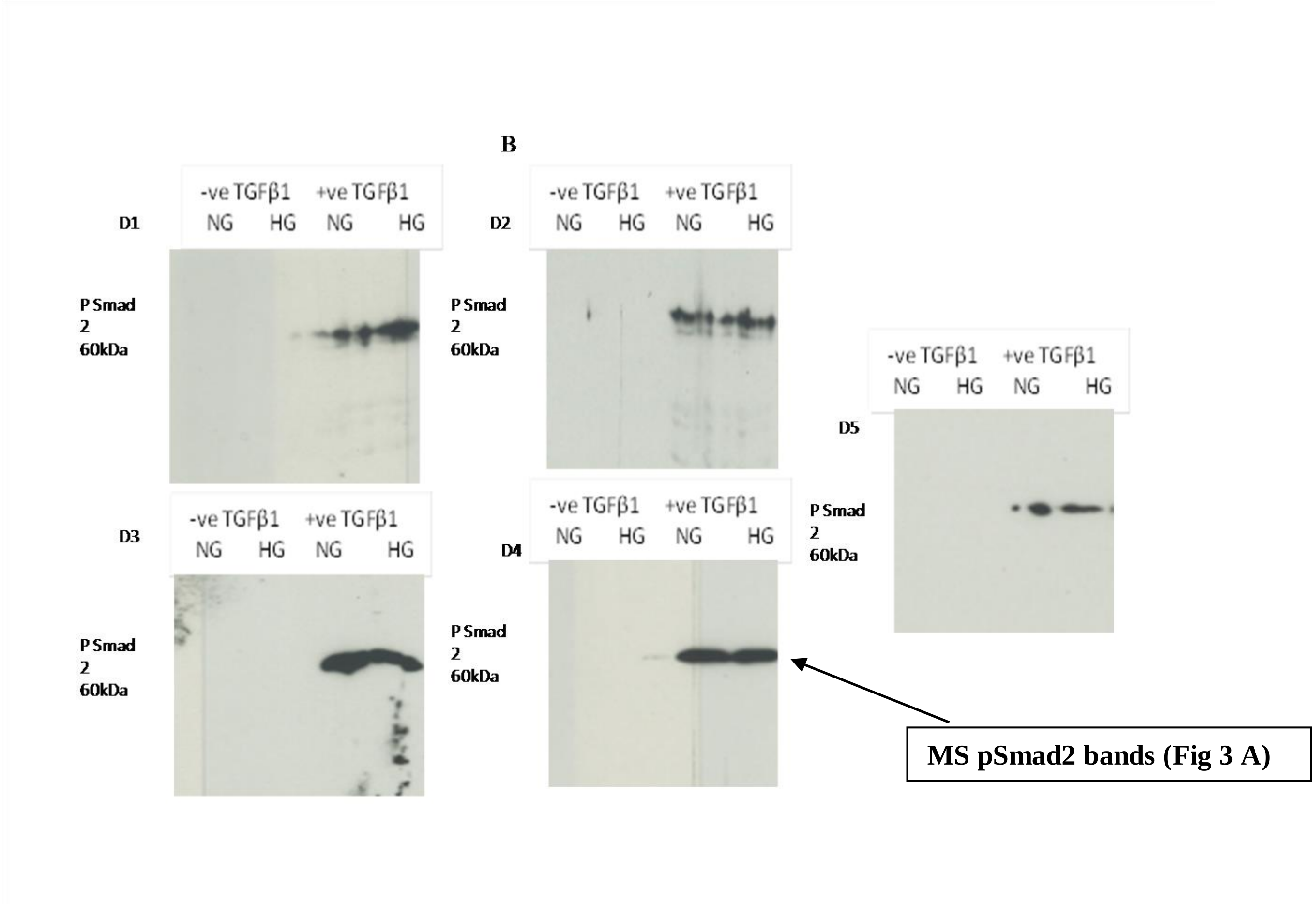

Figure S2. TGFβ1 signaling through Smad2 in Macrophages cultured under NG or HG in other 5 Donors under (D1-D5) the same experimental conditions:

Monocytes were cultured in both NG or HG conditions and stimulated 6 days with IL4/dex.TGFβ1 (10ng/ml) was added. After incubation, the cells were harvested and lysed in a lysis buffer supplemented with phosphatase and protease inhibitors. WB was performed using specific first antibody for the phospho smad 2. WB shows the phosphorylation of smad2 under both NG and HG conditions. The difference in band intensities (protein expressions) shows the different donors responses within the same experimental environment.

Figure S3

MS Figure 3A (Smad1)

MS Smad1 bands (Fig 3 A)

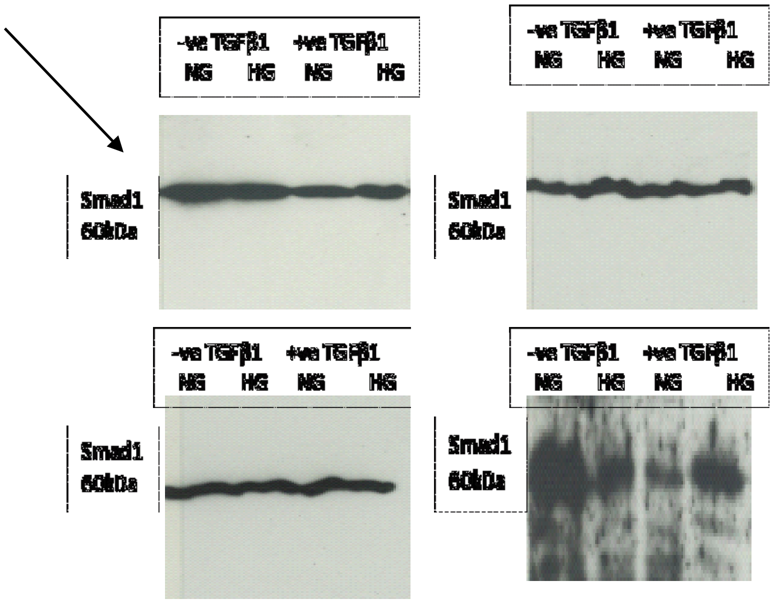

Figure S3. TGFβ1 signaling through Smad 1 in Macrophages cultured under NG or HG in 4 Donors (D1-D4) under the same experimental conditions:

Monocytes were cultured in both NG or HG conditions and stimulated 6 days with IL4/dex.TGFβ1 (10ng/ml) was added. After incubation, the cells were harvested and lysed in a lysis buffer supplemented with phosphatase and protease inhibitors. WB was performed using specific first antibody for the smad 1. WB shows the Un-phosphorylated smad 1 in the same 4 donors. The difference in band intensities (protein expressions) shows the different donors responses within the same experimental environment.

Figure S4

MS Figure 3A (Smad2, 3)

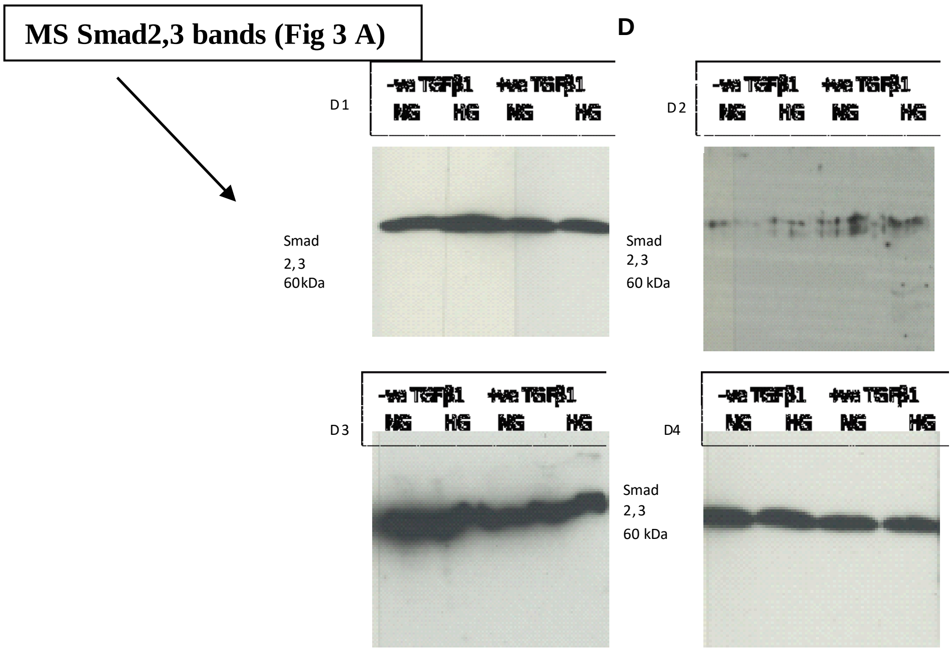

Figure S4. TGFβ1 signaling through Smad2,3 in Macrophages cultured under NG or HG in 4 Donors (D1-D4) under the same experimental conditions

Monocytes were cultured in both NG or HG conditions and stimulated 6 days with IL4/dex.TGFβ1 (10ng/ml) was added. After incubation, the cells were harvested and lysed in a lysis buffer supplemented with phosphatase and protease inhibitors. WB was performed using specific first antibody for the smad 2, 3. WB shows un-phosphorylated smad 2, 3 of the same 4 donors. The difference in band intensities (protein expressions) shows the different donors responses within the same experimental environment.

Figure S5

MS Figure 3A (GAPDH)

MS GAPDH band (Fig 3 A)

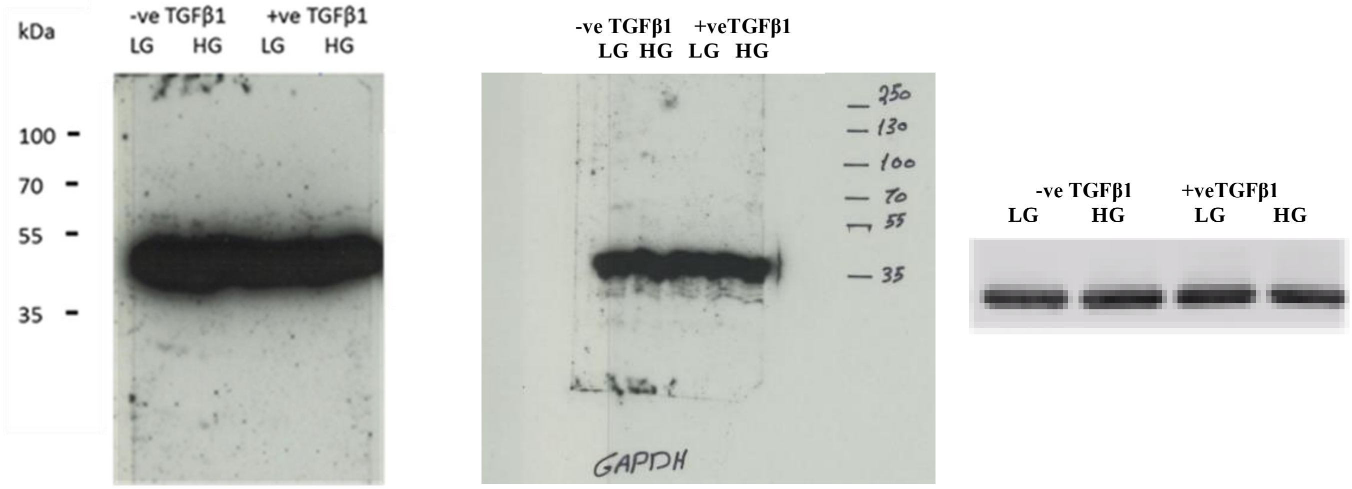

Figure S5. GAPDH in Macrophages cultured under NG or HG in 3 Donors under the same experimental conditions:

Monocytes were cultured in both NG or HG conditions and stimulated 6 days with IL4/dex.TGFβ1 (10ng/ml) was added. After incubation, the cells were harvested and lysed in a lysis buffer supplemented with phosphatase and protease inhibitors. WB was performed using specific first antibody for the GAPDH.

Figure S6

MS Figure 3B

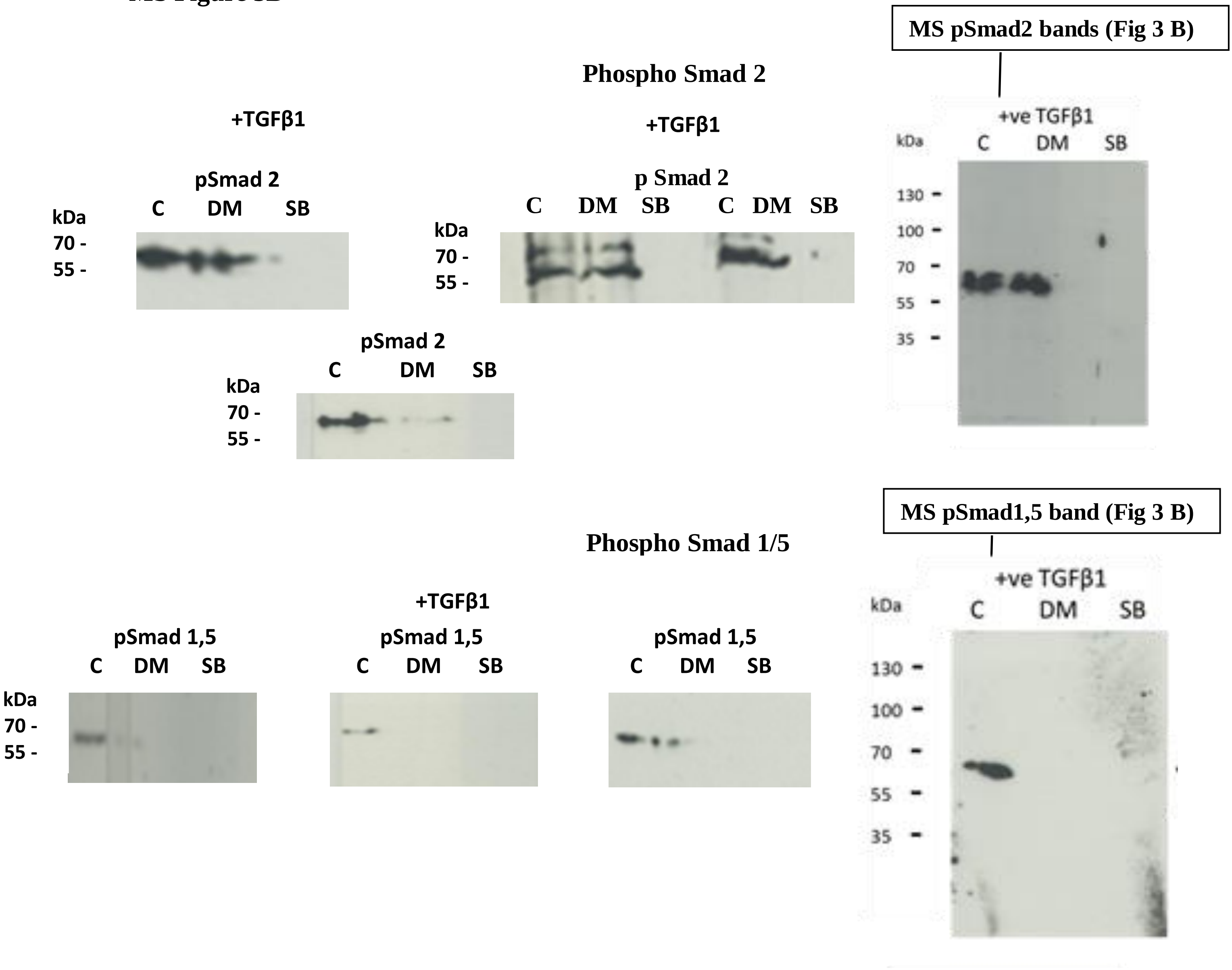

Figure S6. Specific ALK5 inhibitor (SB, 5uM) prevents TGFβ1 signalling through both smad2/smad1, 5 pathways while the specific BMPs signaling inhibitor (DM, 1uM) only prevents smad1, 5 but not smad2 phosphorylation in Macrophages cultured under HG conditions.

Monocytes were cultured under HG conditions and stimulated for 6 days with IL-4/Dex. TGFβ1 (10ng/ml) was added. Specific ALK5, ALK2 inhibitors was added 1h before TGF β1 stimulation. After incubation, the cells were harvested and lysed in a lysis buffer supplemented with phosphatase and protease inhibitors. Western blot analysis revealed inhibition of Smad2 phosphorylation upon inhibition of Alk5 but not Alk1, 2 while smad1, 5 phosphorylation is inhibited by both inhibitors.
